# The return of the Scarlet Pimpernel: cobalamin in inflammation II — cobalamins can both selectively promote all three nitric oxide synthases (NOS), particularly iNOS and eNOS, and, as needed, selectively inhibit iNOS and nNOS

**DOI:** 10.1080/10520290701791839

**Published:** 2008-01-10

**Authors:** Carmen Wheatley

**Affiliations:** Orthomolecular Oncology, 4 Richmond Road, Oxford OX1 2JJ, UK

**Keywords:** Cobalamin, aquacobalamin, methylcobalamin, adenosylcobalamin, glutathionylcobalamin, nitrosylcobalamin, transcobalamins, inflammation, selective promotion/inhibition nitric oxide synthases, nitric oxide, GSNO, tumour necrosis factor alpha, interferon, IRF-1, tetrahydrobiopterin, GTP, arginine, glutathione, heme enzymes, G6PDH, NADPH, succinyl CoA

## Abstract

The up-regulation of transcobalamins [hitherto posited as indicating a central need for cobalamin (Cbl) in inflammation], whose expression, like inducible nitric oxide synthase (iNOS), is Sp1- and interferondependent, together with increased intracellular formation of glutathionylcobalamin (GSCbl), adenosylcobalamin (AdoCbl), methylcobalamin (MeCbl), may be essential for the timely promotion and later selective inhibition of iNOS and concordant regulation of endothelial and neuronal NOS (eNOS/nNOS.) Cbl may ensure controlled high output of nitric oxide (NO) and its safe deployment, because: (1) Cbl is ultimately responsible for the synthesis or availability of the NOS substrates and cofactors heme, arginine, BH_4_ flavin adenine dinucleotide/flavin mononucleotide (FAD/FMN) and NADPH, via the far-reaching effects of the two Cbl coenzymes, methionine synthase (MS) and methylmalonyl CoA mutase (MCoAM) in, or on, the folate, glutathione, tricarboxylic acid (TCA) and urea cycles, oxidative phosphorylation, glycolysis and the pentose phosphate pathway. Deficiency of any of theNOS substrates and cofactors results in ‘uncoupled’ NOS reactions, decreasedNO production and increased or excessive O_2_^−^, H_2_O_2_, ONOO^−^ and other reactive oxygen species (ROS), reactive nitric oxide species (RNIS) leading to pathology. (2) Cbl is also the overlooked ultimate determinant of positive glutathione status, which favours the formation of more benign NO species, s-nitrosothiols, the predominant form in which NO is safely deployed. Cbl status may consequently act as a ‘back-up disc’ that ensures the active status of antioxidant systems, as well as reversing and modulating the effects of nitrosylation in cell signal transduction.New evidence shows that GSCbl can significantly promote iNOS/ eNOS NO synthesis in the early stages of inflammation, thus lowering high levels of tumour necrosis factor-a that normally result in pathology, while existing evidence shows that in extreme nitrosative and oxidative stress, GSCbl can regenerate the activity of enzymes important for eventual resolution, such as glucose 6 phosphate dehydrogenase, which ensures NADPH supply, lactate dehydrogenase, and more; with human clinical case studies of OHCbl for cyanide poisoning, suggesting Cbl may regenerate aconitase and cytochrome *c* oxidase in the TCA cycle and oxidative phosphorylation. Thus, Cbl may simultaneously promote a strong inflammatory response and the means to resolve it.

## Introduction

A Scarlet Pimpernel for the Resolution of Inflammation? [1] proposed that vitamin B12, cobalamin (Cbl), in all its various forms, is central to the effectiveness of the immune inflammatory response, and that its deficiency, chronic, functional or ‘compartmental’, may largely contribute to the aetiology of systemic inflammatory response system (SIRS)/ sepsis/septic shock, as well as autoimmune disease, central nervous system (CNS) disease, cancer, in particular haematological malignancy [2], and the progression of AIDS. The hitherto unexplained elevation of Cbl carrier proteins, the transcobalamins (TC I, II and III), their receptors, and TC unsaturated B12 binding capacity (UBBC) in trauma, infections, chronic inflammatory conditions [3–9] and some cancers [2,9–13] was seen to signal a central need for Cbl as a principal regulator of inflammation. The initial hypothesis proposed that Cbl might exert a pivotal effect on inflammation via regulation of the redox sensitive transcription factor, NFκB [14], which determines the expression of a diversity of genes encoding mediators of the pro- and anti-inflammatory phases of the immune response: cytokines, chemokines and inducible enzymes, principally, cyclooxygenase (Cox II), inducible nitric oxide synthase (iNOS) [15] and heme-oxygenase (HO-1) [16]. Regulated expression of such genes by NFκB, a family of rel protein homo- and heterodimers (RelA/p65, RelB, cRel, p50, p52), ultimately determines cell survival or proliferation, tissue repair and apoptosis. Evidence for five interrelated mechanisms by which Cbl might regulate NFκB was put forward: (1) hormone-like regulation of tumour necrosis factor-α (TNFα), through scavenging of excess nitric oxide (NO) by Cbl, as well as through the selective inhibition by Cbl, in tandem with gluthathione, of iNOS; (2) Cblquenching of NO radicals (RNIS) and reactive oxygen species (ROS), enhanced by Cbl's glutathione (GSH) sparing/promotional effect; (3) Cbl promotion of acetylcholine synthesis, central to the neuro-immune cholinergic anti-inflammatory pathway; (4) Cbl's promotion of cellular energy and respiration via the tricarboxylic acid (TCA) cycle and oxidative phosphorylation; (5) a bacteriostatic role of the TCS released by neutrophil secondary granules during phagocytosis, which also appears to modulate the inflammatory response [1].

Recent *in vitro* explorations of some aspects of the original hypothesis have shown that, at least in the pro-inflammatory phase, Cbl does not inhibit NFκB [17], and that indeed certain Cbls have a slightly promotional, although not statistically significant, effect on NFκB [17]. What direct/indirect effect Cbl may have on NFκB in the anti-inflammatory resolution phase of the immune response remains to be explored in a temporal *in vivo* model [18]. However, a totally novel *in vivo* finding of a strong promotional effect of Cbl, particularly glutathionylcobalamin (GSCbl), on iNOS, with simultaneous supportive promotion of endothelial NOS (eNOS), in the early stages of inflammation [17] (further corroborated by an inversely related suppression of the glucocorticoid, annexin-1, and lower, well-regulated levels of TNFα) [17], may be consistent with one of the original hypotheses, that the ubiquity of Cbl and GSH is due to their mutual regulation of NO, in a continuous scavenger–donor redox dance [1]. Because NO produced by iNOS can ultimately inhibit iNOS [19,20] in the resolution of inflammation, as wellas NFκB at its conclusion [21,22], a direct promotional effect of Cbl, particularly GSCbl, on iNOS induction [17] would mean that Cbl does ultimately regulate NFκB, indirectly, via NO regulation. If this is so, Cbl status could be the fulcrum on which the entire immune system turns. The Return of the Scarlet Pimpernel will attempt to explore how Cbl might act as both a timely selective promoter and a selective inhibitor of iNOS, as well as a key regulator of all three NOS in general.

## ‘They seek him here. They seek him there…’ Cbl's multiple forms and multiple roles

**Figure 1.**
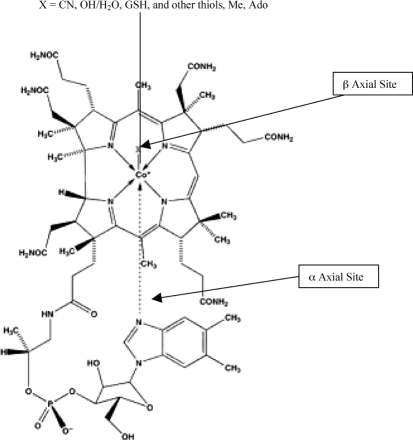
Structure of vitamin B_12_ and its derivatives.

Cbl, C_63–65_H_88_O_14_N_14_PCo, vitamin B12 [23,24], a red crystalline, water-soluble substance (molecular weight 1357 kDa), comprises various polycyclic compounds, with a central cobalt atom set within a planar, tetrapyrrole (corrin) ring, that resembles that of the porphyrin of heme, except that it is less symmetrical. The upper β axial cobalt ligand is variable and can combine with H_2_O, OH, CN, GSH and other thiols, and with Me and Ado to form the coenzymes, methylcobalamin (MeCbl) and 5^1^′-deoxy-5-adenosylcobala-min (AdoCbl) [10]. The latter two have a unique, covalent carbon–cobalt bond that gives Cbl its remarkable chemical and biological reactivity, and makes it one of the most potent physiological compounds, with a daily requirement of only 1 μg. The lower α axial ligand for the principal forms of the vitamin is a ^5–6^dimethylbenzimidazole, ‘false’, nucleotide base (DMBI) (Figure 1). Cbl is nature's most complex non-polymer molecule and the most complex of the vitamins and enzymatic cofactors known to date. It is synthesized by bacteria both in the soil and in the lumen of ruminants. Humans must derive Cbl from their diet, chiefly liver, kidneys, red meat, oysters, egg yolk and yeast extract. Absorption from food is also complex, as it involves the binding of Cbl in food by the Cbl transport protein TCI in saliva, gastric acid to separate Cbl from protein, and intrinsic factor in the ileum, as well as the transport protein, TCII [25]. In the circulation there are, in fact, three transport proteins, TC I, II and III, with separate functions [26]. Cbl also assumes different forms, the two principally known being MeCbl (75–90% of the body pool of circulating Cbl, transported chiefly on TCI) and the coenzyme AdoCbl (10–25% of endogenous Cbl, transported chiefly on TCII). TCIII appears to remove Cbl analogues or corrinoids, and cyanocobalamin (CNCbl) from the tissues and circulation and take them to the liver for excretion in bile, as corrinoids seem to interfere with the function of Cbl, whereas CNCbl, found mostly in the lungs of smokers [11], is probably an excretory, detoxification product and is functionally inert. Cbl, which enters the circulation as OHCbl/H_2_OCbl, is transported by TCII, via the TCII endocytosis ion channel receptor (TCIIr), into all tissues and cells, where, after TCII degradation in the lysosomes, it is converted to MeCbl and AdoCbl, and largely retained for use intracellularly [27], although some is exported on TCII and TCIII [28]. MeCbl acts in the cytosol, AdoCbl in the mitochondria. Synthesized primarily by granulocytes, high concentrations of TCI are found in the reticuloendothelial system, in neutrophils, and in the liver. TCI is largely confined to the circulation, perhaps as a mobile store of Cbl (to complement the larger, long-term storage of Cbl as MeCbl and AdoCbl in the liver and the pool of free Cbl in the kidneys). TCI does not have a specialist receptor, unlike TCII. Instead it is taken into the cell via a multipurpose receptor, the asialoglycoprotein receptor, a liver-specific protein [29]. TCII, a low molecular weight glycoprotein (43 kDa), with β globulin mobility, delivers MeCbl, AdoCbl and OHCbl/ H_2_OCbl and other Cbls to the tissues. However, there is clearly some form of communication and flexibility between the TCS: TCII carries the larger fraction of Cbl present in portal vein blood than in hepatic and axillary vein blood. In disease, TCII sometimes holds the bulk of Cbl present in peripheral blood [30], suggesting Cbl transfer from TCI as needed. High concentrations of TCI are also found in extracellular fluids: milk, saliva, tears, semen, amniotic and spinal fluid. Moreover, although every DNA synthesizing cell in the body contains receptors for TCII, the principal TC in tissues, there are nonetheless fine gradations of all three TCS present in cells, varying continuously in amount and intracellular location, according to the cell type and stage of maturation [31].

The elegance and ubiquity of such a fine-tuned and flexible Cbl delivery system is further adapted during inflammation with a rapid response in the liver and granulocytes to produce marked elevations in the TCS, either I and/or II [3], their receptors [4] and UBBC [4,5]. This is true for both chronic inflammation [for example, rheumatoid arthritis (RA) [6], systemic lupus erythematosus [7], diabetes, Crohn's disease [8]] and acute inflammation, including cancer-associated inflammation, trauma and infections [4,5]. The TCs and their UBBC are also increased in Cbl deficiency [10], notably in the immune-compromised (AIDS [23,32] and cancer patients [10–13,23,32]) and the elderly [33] (who have a median of 40.5% Cbl deficiency) [34], the same two groups who are also most susceptible and likely to succumb to SIRS/sepsis/severe sepsis and septic shock [35]. Although TCS have been labelled ‘acute phase response proteins’, the possible significance of this has been either overlooked and no crisis function ascribed to them, or, as in the case of TC elevations in cancer, they have been negatively interpreted.

## The mysterious go-between: GSCbl and B12 coenzymes

The conversion of aquacobalamin (H_2_OCbl) on cell entry to the coenzymes MeCbl and AdoCbl is not straightforward. It apparently proceeds via the formation of the unusually stable intermediate [36,37] GSCbl, the product of H_2_OCbl+and excess reduced GSH only [38–40].GSCbl is believed to be a major form of intracellular Cbl [41], although this has not yet been proven unequivocally, and GSCbl's exact biological role, other than as a principal intermediate on the pathway to MeCbl and AdoCbl coenzyme formation [42], remains to be fully explored. As befits its role as intermediate for MeCbl and AdoCbl formation, GSCbl may be a true go-between and also act independently, both in the cytosol and mitochondria. (The possible significance of such GSCbl flexibility will be discussed in the Hypothesis section on the GSH, Cbl, NO triad relationship.) Recent chemical discoveries about GSCbl may point to its potential biochemical significance. The observed rate constant for the formation of GSCbl increases with decreasing pH, reaching a limit value at pH<6. Conversely, the equilibrium constant for the formation of GSCbl from H_2_OCbl+and GSH in the pH range 4.50–6 increases with increasing pH [38]. Intracellular pH is tightly regulated. However, unlike extracellular pH, which is a constant 7.3–7.4, intracellular pH varies according to location and vocation, from 7–7.3 in the cytosol to 5–6 in the endosome, with the pH also notably lowering in the lysosome, phagosome, and secretory granules to 5–6 or 5.5 [43]. Thus, I would propose that the formation of GSCbl is so set up that, as in degrees of inflammation intracellular pH becomes even more acidic, GSCbl is formed increasingly rapidly, but in controlled amounts, presumably to conserve its steady availability over the crisis period. Then, as inflammation is resolved and cellular pH returns to normal, GSCbl formation equilibrium increases and the rate constant decreases. Nevertheless, even at the normal cytosolic pH of 7.4 and the normal body temperature of 37°C, conversion of H_2_OCbl+to GSCbl will occur almost instantaneously, with a half-life of 2.8 sec for the reaction with 5 mM GSH [38]. (Levels of GSH in cells can range up to 10 mM.)

It may be an index of the previously posited special relationship between Cbl and GSH that the much larger than expected formation constant for GSCbl shows that thiolate forms of GSH are the first identifiable Cbl ligands to approach the remarkably high binding affinity of CN to H_2_OCbl [38]. (Based on the pH dependence of KobsGSCbl in the pH region 4.5–6, an estimate of *K* GSCbl in the order of 5 × 10^9^ M^−1^is the closest formation constant to that of CN for H_2_OCbl≈10^14^M^−1^ [38].) It is pertinent also that AdoCbl formation is four times greater from GSCbl than from H_2_OCbl or CNCbl [42]. In coenzyme formation, GSCbl is postulated as interacting directly with the active sites of methionine synthase (MS) or methylmalonyl CoA mutase (MCoAM), and after reduction to Cob(I)alamin it is believed it may react respectively with s-adenosylmethionine (SAM) or adenosine triphosphate (ATP) to form enzyme-bound MeCbl and AdoCbl [38]. It is not known whether some GSCbl is protein bound intracellularly, or whether any of it is exported on TCII and TCIII, as small amounts of MeCbl and AdoCbl are. However, both MeCbl and AdoCbl are largely protein bound, and in an unexpected manner. A huge surprise for B12 chemists when the crystal structures of the two enzyme-bound cofactors were elucidated, was that the DMBI, in both enzyme-bound MeCbl and AdoCbl, is ‘base-off’, that is, the DMBI is no longer co-ordinated to the cobalt, which is instead liganded or base-on to the imidazole Nɛ2 in one of the two proteins' histidine residues (histidine 759 and A610 for MS and MCoAM, respectively). The DMBI had been expected to play a crucial allosteric role in the enzymes' catalysis, but instead is confined to functioning as an anchor sunk into a deep hydrophobic pocket in both enzymes [1,44–46]. Some 16 Cbl-dependent enzyme reactions are known to date, of which only two, possibly three, the latter is controversial [1,47,48], occur in man. Yet, these two Cbldependent enzymes control key metabolic pathways, whose far-reaching relationships and often ‘hidden hand’ (of the Scarlet Pimpernel) consequences ensure the protection of every organ and system of the body.

As a methyl donor, MeCbl, via MS, reduces homocysteine to methionine, whichthen combines with ATP to form SAM, and ensures good methylation of DNA, RNA, protein, and successful DNA replication [2,5,49]. (Therefore, Cbl, as well as folate, status should be critical for cancer chemoprevention [2,5,49,50].) MS also reduces N^5^-methyltetrahydrofolate (NMTHF) to H_4_folate (THF), thus ensuring its bioavailability for purine and δTMP synthesis [2,50]. AdoCbl, via MCoAM, mediates the isomerization of methylmalonyl-CoA to the energy-rich thiol ester, succinyl CoA, the formation of which AdoCbl shares with α-ketoglutarate (αKG). This means that Cbl acts at a critical stage in the Krebs or TCA cycle, as succinyl CoA represents a metabolic branch point wherein intermediates may enter or exit the cycle, leading ultimately to the release of guanosine triphosphate (GTP), a source of energy in gluconeogenesis and protein synthesis, and, in collaboration with the electron transport oxidative phosphorylation chain, to release of ATP. Succinyl CoA may also be converted to succinate or condensed with glycine to form δ-aminolevulate, the initial step in porphyrin biosynthesis. The conversion of methylmalonyl-CoA to succinyl CoA is alsoimportant for the catabolism of valine, isoleucine, methionine; the pyrimidine DNA-specific nucleobase, thymine; odd-chain fatty acids; and degradation of the side-chain of cholesterol [51]. Cbl is thus essential for cellular respiration and energy, and both protein synthesis and catabolism.

**Figure 2.**
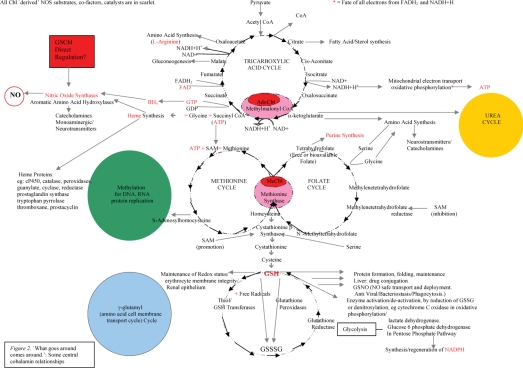
‘What goes around comes around’: some central cobalamin relationships.

The web of complex inter-relationships sustained by the two mammalian Cbl coenzymes is illustrated in Figure 2. It may be seen from this that degrees of Cbl deficiency may result in malfunction on many levels. Perhaps then, it is not surprising that, although some remain sceptical, the Cbl chemical, biochemical, medical literature [52], including clinical case histories, now supports claims, and increasing evidence, for the efficacy of Cbl in everything from cancer [2,52], heart disease [52], autism [52], Alzheimer's disease [52], multiple sclerosis [52] and other neurological conditions [52], AIDS [52], SIRS/sepsis/septic and traumatic shock [1], infertility [52], depression [52], circadian rhythm disorders [52], autoimmune disease [52], chronic fatigue syndrome [52], eczema and other skin conditions [52], allergies [52], and, not least, growth and megaloblastic anaemia, as Cbl is critical to haemopoiesis [52]. Patents granted or applied for include evidence that Cbl promotes anti-inflammatory HO–1 while lowering inflammatory arachidonic acid metabolites, such as 12(R)-HETE and 12(R)-DiHETE (USP 5,674,505): also that Cbl, alone or combined with interferon or chemotherapeutic agents, is effective in viral, proliferative and inflammatory disease, including hepatitis C and B, herpes, vesticular stomatitis, autoimmune encephalomyelitis, multiple sclerosis and astrocyte gliomas (USP application 2005/0163751 A1). This pleiotropic character of Cbl's effects is curiously reminiscent of that of NO. Is this just a coincidence?

## The NOCbl controversy

The fact that Cbl has some kind of rapid physiological impact on NO, which might prove beneficial in pathology involving unresolved inflammation, is suggested by various reports dating back to 1991. Large intravenous doses of OHCbl significantly increased systemic vascular resistance in normal conscious dogs [53]. The relaxation of isolated vascular and visceral smooth muscle induced by NO and NO donors was reversed by OHCbl [54]. Mice exposed to lipopolysaccharide (LPS) and given either CNCbl or OHCbl had a 30 and 40% increased survival, respectively (with zero survival for the control group) [55]. OHCbl also blocked NO-mediated inhibition of leukaemia cell proliferation [56]. Such data has led to the popular view of Cbl's role in inflammation as a scavenger for excess NO, supposedly combining with it to form nitrosylcobalamin (NOCbl) [55–58]. Although studies show this can happen, in a reversible manner, at the point of an electrode [59], the existence of NOCbl has been much disputed by B12 chemists [56,60–62], and an alternative mechanism for Cbl's inactivation of NO has been proposed, as NOCbl is not thought to be a stable enough complex to account for the inhibition/blockade of NO's biological effects by Cbl. Based on the observation that superoxide can, under certain conditions, lead to the rapid inactivation of NO, it has been proposed that Cbl (III)O_2_^−^, a superoxide species of Cbl that rapidly and spontaneously regenerates in aerobic solutions, interacts with NO by forming ONOO^−^(peroxynitrite) in a cyclic mechanism for its rapid inactivation [60]. Although not implausible, this mechanism remains hypothetical, as Cbl (III)O_2_^−^ is too unstable and difficult to purify for direct dose–response studies. A more recent study that apparently established rapid interaction between NO and the Cbl of MS, in the Co^+1^ state, Cbl (I), based on spectroscopic analysis, proposed that NO, at physiologically normal concentrations, may regulate carbon flow through the folate pathway by inhibiting MS and decreasing rates of methionine, serine, and de novo purine nucleotide synthesis. In this cell culture *in vitro* model, homocysteine (Hcy)[63] was seen to act as an inhibitor of NO, a perhaps surprising finding given that NO is normally seen as anti-atherogenic, yet by inactivating MS, NO raised the levels of supposedly pro-atherogenic homocysteine. This hints at a complexity in the NO/ Cbl relationship outside the study model's parameters.

Moreover, much of the data cited above for direct NO/Cbl interaction is handicapped by being largely based on purely chemical studies, or in vitro or isolated tissue studies that necessarily omit full physiological complexity. The view that Cbl just scavenges NO has always seemed too simplistic to the writer of this hypothesis. Combining with NO in a physiologically consequential way is more the province of heme and non-heme iron, O_2_, superoxide, GSH and other thiols [64]. If NOCbl has a physiological role, then maybe it is a very transient, possibly negligible, one, possibly less negligible in pathology, given the proposed impact of NO on MS [63]. Nevertheless, it is still a question whether NOCbl exists at all endogenously. So far NOCbl has not yet been detected *in vivo* (although it has been given exogenously to mice as an effective anti-cancer agent [65]). Indeed, if Cbl were capable of continuous competition with heme or non-heme iron or GSH as a successful rival for NO, it might prove dangerous to life. Whereas, the rodent, large mammal and human clinical literature demonstrates that Cbl in high, even extremely high, supra-physiological doses is a life restorer in perilous situations, even capable of resurrecting the dead [66]. (‘Des souris en état de mort apparènte parairent être réanimées par iv. 250 mg/kg OHCbl, sans autre mésure’ after cyanide poisoning [66].) Apart from its traditional use as the treatment for subacute neuronal degeneration of the spinal cord and pernicious anaemia, a discovery that in its day was equivalent to finding a cure for cancer, Cbl has been shown to work safelyin a varietyof animal models: mice, rats [55], guinea-pigs [67], dogs [68], baboons [69] and in a variety of extreme situations, from the trauma of radiation [70] and electrocution [71] to fatal injury [68], anaphylactic [67] and septic shock [55]. In addition, Cbl has been used successfully for over 40 years in the intensive care unit in France (but also latterly in Spain, Italy, Germany, Hong Kong and China). The French case literature [72–76] documents near-miraculous recoveries from cyanide poisoning with the use of OHCbl as an antidote, given in extraordinarily high doses of 4–5 g, sometimes repeated on consecutive days. At these doses, the only side-effects reported were a transient urticaria or red rash. Cyanide victims thus rescued are documented as recovering consciousness and cardiovascular function within 30 min of Cbl infusion, and walking out of the intensive care unit within 2 days, with liver and other vital organs intact. This is not the normal intensive care unit experience with such or similar extreme conditions, and may not therefore, this hypothesis maintains, be solely ascribed to the binding of cyanide by Cbl. The same supra-physiological 5 g dose of OHCbl given to normal heavy smoking volunteers produced only modest transient rises in blood pressure and slight transient bradycardia [77], conditions that might be welcomed as side-effects in sepsis or shock treatment. Indeed, if Cbl were just an NO mop, it might be expected to cause dangerously high blood pressure and persistent vasoconstriction. Instead, few drugs can hope to emulate Cbl's proven pharmacological safety profile [53,72–77].

So the Cbl/NO relationship has to be more complex and interesting than the crude idea of Cbl as just an NO mop. It is much more plausible, given the safety and efficacy literature, that Cbl should exert a central control over NO, in part through the regulation of all three NOS and through selective promotion and inhibition [1,78] of iNOS, as and where it is needed. Because recent in vivo studies at the William Harvey Institute [17], with other corroborative markers, show quite clearly that high-dose OHCbl, and particularly GSCbl, promote iNOS mRNA in the early stages of LPS-induced inflammation, and because mice given high-dose Cbl to treat LPS-induced sepsis show remarkable survival [55], it is possible that Cbl first promotes iNOS NO production and later, in the resolution phase of inflammation, inhibits it, perhaps over and above iNOS inhibition by NO itself. Moreover, it may be that the high levels of iNOS NO production apparently promoted by Cbl, particularly by GSCbl [17], in the pro-inflammatory phase are essential for regulating inflammation and signalling entry into the resolution phase. The question is how?

## Hypothesis

A well-regulated, successful immune inflammatory response is biphasic [79,80]. A pro-inflammatory phase, entailing the tyrosine kinase phosphorylation cascade (MAPK) and the activation of key transcription factors, such as STAT-1, NFκB, AP-1, Sp1, IRF-1, which up-regulate the production of inflammatory cytokines, principally, TNFα, interleukin 1β, interleukin-6, interferons α, β and γ, chemokines, adhesion molecules, growth factors, proteases, inducible enzymes such as HO-1, Cox II, iNOS, phospholipase A_2_ as well as prostaglandins, particularly PGE-2, and other lipid mediators, such as platelet activating factor, leukotrienes, thromboxanes, and tissue factor, which activate the extrinsic coagulation cascade. Such pro-inflammatory factors radically change the redox environment at the site of inflammation, particularly in the macrophages [81] and neutrophils [82], so that it becomes more oxidant. Increased oxidative products, such as superoxide (O_2_^−^), singlet oxygen, hydroxyl radicals, NO and its species, interact to form other potentially lethal species, such as H_2_O_2_ and ONOO^−^. These free radicals collectively play vital roles in the immune response, acting both as signalling/acute response activation agents and as cytotoxic agents [82–86]. For maximum lethality to the invader and minimum damage to the host they must be deployed in a focussed and balanced manner [79], which has a natural time limit and often a distinct, spatially confined, or discrete intracellular location [86]. Key antioxidant enzyme systems up-regulated by decreasing pH, ensure that balance, focus, time and spatial limitation, signal specificity and efficacy are maintained [84–88]. Superoxide dismutase (SOD), for example, removes excess superoxide using different catalytic transition metals, Cu, Zn, Mn, in differing cell environments — mitochondria, cytosol, phospholipid membrane [89]. Catalase scavenges H_2_O_2_ [90]. GSH [91], a major reductant and detoxifier, continuously recycled by selenium-dependent GSH peroxidase (GPX), which also scavenges H_2_O_2_, is subsequently reduced again by GSH reductase [91], while glutathione-S-transferase is also up-regulated by the transcription factor AP-1, which is under direct control by NO [92]. HO-1 breaks down heme to biliverdin [16], which in turn yields the antioxidant, bilirubin [93]. Heme peroxidases, such as myeloperoxidase, consume H_2_O_2_ in phagocytes and Cox II [90]. Glucose 6 phosphate dehydrogenase (G6PDH) produces a steady stream of the reducing equivalent NADPH, needed for key enzyme catalysis [94]. Thus, by a complex interplay of phosphorylating/redox-sensitive signalling/ response systems, the inflammatory phase of the immune response is normally self-limiting. But rather than giving way in a see-saw manner to the resolution phase, the evidence so far suggests that the anti-inflammatory phase is engaged early on, but, initially, at a much lower level, its activity increasing as the peak of inflammation is reached and then declines. This may depend on early parallel activation of the cholinergic immune pathway [95], in tandem with the sympathetic/adrenal neuro-endocrine pathway, involving the release of glucocorticoids, such as Annexin 1 [96], and the catecholamines dopamine, epinephrine/norepineph-rine; also the deployment of ‘good’ eicosanoids, such as the ω-3 fatty acid, eicosapentanoic acid, known to suppress TNFα and interleukin-1 [97], and various oxidized derivatives of eicosapentanoic acid, neuro-protectins [98], resolvins [99], lipoxins [100]. Eventually antiinflammatory cytokines interleukin-4, interleukin-10 [101], interleukin-13, and growth factors, epidermal growth factor (EGF) and transforming growth factor β1 (TGFβ1) [102] are fully expressed, preceded by the inhibition of iNOS and a change in sense of pro-inflammatory factors such as nuclear translocated NFκB, Cox II and interleukin-6, which can paradoxically also signal repair and resolution in due course [103].

In pathologies of unresolvable inflammation, chronic or acute, such as multiple sclerosis, RA, or sepsis, the redox balance is lost, either locally or systemically, with devastating results in the latter case. In sepsis, endogenous antioxidant enzyme systems can be depleted within hours, with a 46–83% loss of activity in SOD and GPX, just 12 hours into sepsis, and a 52% reduction in the somewhat more resistant liver catalase [104]. With little to keep it in check, a fireball of reactive oxygen intermediates (ROS) and nitric oxide species (RNIS) fuels the pro-inflammatory phase as it works in a feed-forward, continuously amplifying loop of widespread endothelial and epithelial damage; a build-up of fibrin impeding circulation in the microvasculature: unresponsive hypotension; and increasing O_2_^−^ and ONOO^−^dramatically impairing cellular respiration and energy production, through increasing inhibition of aconitase in the Krebs cycle and complexes I to IV in the mitochondrial oxidative phosphorylation, electron transport chain [105], all lead, if prolonged, to cell death and eventual multi-organ failure.

It is commonly thought that the chief cause of such scenarios is NO overproduction by iNOS [106–108], a view based largely on numerous *in vitro* studies, often with exogenous NO donors, (studies, which by definition, omit the effect of potential systemic iNOS/nNOS modulation systems, such as the increase in circulating and tissue entry TCS,) or on murine studies that do not discriminate between iNOS mRNA and the potential for variability in its redox products [109], or that use even less specific evidence, such as serum/ urine nitrite/nitrate. Although there is not much large mammal or human data for it [106], such evidence as there is for this view, seems largely based on plasma and/or urine measurements of nitrite, and nitrate, in RA or septic shock patients, for example [106–108], as direct NO and NOS assays *in vivo* are fraught with difficulty [110]. But this evidence is equivocal, as nitrite and nitrate do not necessarily indicate simply formation of NO, but can equally well be derivatives of RNIS, such as ONOO^−^, or its protonated species, HNO [111]. Indeed, a recent study of nitrite/nitrate excretion in the serum, urine, saliva and tears of RA and healthy age-matched controls, both on a low nitrate/nitrite diet, found no significant differences, or relationships [112]. Moreover, nitrite and nitrate are also products of protein catabolism [113], which is dramatically increased in pathologies of acute unresolved inflammation, such as burn injury and sepsis [114]. The fact that NOS/ iNOS inhibitors can attenuate the hypotension of sepsis is also non-specific evidence for putative NO damage. It can equally well be argued that iNOS inhibitors also inhibit the production of RNIS, which may be much more likely candidates as a cause of hypotension than NO. iNOS inhibitors will also considerably reduce the production of TNFα and interleukin-1, and this too may be material, as high dose administration of TNFα is known to produce lethal hypotension [115], which may have been automatically attributed to assumed high NO. Moreover, there is evidence that levels of NO have a direct regulatory correlation to levels of TNFα. In a murine model using *Staphylococcus B*, NO inhibitors increased sustained release of TNFα (and interferon-γ), which increased enterotoxin toxicity of *Staphylococcus B* [116]. Anti-interferon-γ monoclonal antibodies were more effective at NO reduction than anti-TNFα monoclonal antibodies, but together they produced total NO inhibition [116]. This suggests the existence of a regulatory loop by which NO inhibits the production of TNFα/interferon-γ, which induces its own synthesis [81,117,118]. This TNFα/NO relationship has been observed elsewhere. TGFβ-1, usually expressed in the resolution of inflammation, is a potent suppressor of NO *in vitro* and *in vivo*, and TGFβ-1 transgenic mice exposed to LPS show blunted production of NO, but an eight-fold higher production of TNFα, as opposed to wild controls, with consequent increased mortality [119]. The recent studies at the William Harvey Institute also demonstrate this high NO–lower TNFα relationship, as a result of high-dose Cbl administration in mice exposed to LPS [17]. Because Cbl has also been shown to exert direct hormonal-like regulation of TNFα [120], it is reasonable to conclude that such Cbl/TNFα regulation is the result of Cbl/NO regulation. The pleiotropic transcription factor Sp1, which directly promotes transcription of the TCII gene [27] (up-regulated in inflammation) and of iNOS expression [81], is also involved in regulating TNFα transcription via the Sp1 binding site of its promoter, in response to iNOS NO production [121]. Sp1 is moreover involved in the regulation of anti-inflammatory TGFβ [122], and epidermal growth factor [123], the latter known to be directly Cbl status dependent [120]. Thus, Sp1, the TC-Cbl carrier promoter, is responsible for parallel activation of pro-and anti-inflammatory factors, just as this hypothesis proposes Cbl may be. The emphasis, however, is on Sp1/Cbl-NO regulation of TNFα, not suppression. There must be a necessary right level of TNFα for a successful immune response, as anti-TNFα antibodies in the clinic increase mortality [124]. Similarly too, although it may seem an old, discarded paradigm, there may be a right level of NO, higher in relation to TNFα, for a successful immune response outcome. Hence, even setting aside the detrimental impact of non-selective NOS inhibitors on eNOS, this is an additional explanation for the negative outcome of iNOS suppression in sepsis with increased mortality in the clinic [106], prefigured in animal models. iNOS knockout mice treated with LPS showed no significant survival over the wild-type [125]. Other iNOS −/− mice showed no defence against Gram-positive bacteria, and equal mortality and vital organ damage as the wild-type [126]. Furthermore, macrophages derived from these iNOS −\− mice failed to restrain the replication of *Listeria* monocytogenes *in vivo* and lymphoma cells *in vitro*. Since iNOS is primed to be inhibited by NO feedback [19–21], it should in effect be inhibited by putative NO overproduction in sepsis. Clearly, it is not. So, NO overproduction seems less plausible as the source of trouble.

This hypothesis, then, proposes a contrary scenario: it is not NO per se that is the problem in unresolved inflammation. Rather, the problem may be a malfunction of iNOS, resulting from degrees of mild, subclinical or ‘functional’ Cbl deficiency, which might also involve ‘compartmental’ Cbl deficiency, with the local inactivation of one of the two Cbl coenzymes, MS in the CNS, for example [127], allied perhaps to other factors such as age, immune compromise, poor general nutritional status, and/or iNOS, TNFα, interferon, platelet activating factor, TC, gastric atrophy, or other, genetic polymorphisms. A polymorphism, in the iNOS gene, for example, which promotes greater NO production, has been shown to confer greater resistance to malaria [128]. Polymorphisms in the NRAMP1 gene, an intracellular NO chaperone, may also impact significantly on immune resistance [129]. [As an epidemiological aside, US statistics show a significant increase of 139% in sepsis diagnoses since the 1980s, with the increase especially notable in patients over 65 years of age (162%) [106], the very group notable for a 40.5% median Cbl deficiency [32]. What is also notable since the 1980s is the introduction and widespread, often indiscriminate, use of proton-pump inhibitors and H_2_-blocker drugs, both of which interfere seriously with acid-dependent Cbl absorption [130,131]. The effects of the latter may be amplified by a decline in consumption of liver and kidneys, the post-war generation's staple, and, latterly, red meat and eggs, all key sources of Cbl.]

The numerous studies that show a detrimental effect of iNOS activation on pro-inflammatory factors do not appear to have taken into consideration that when iNOS, the least tightly ‘coupled’ NOS isoform, malfunctions it can catalyse reactions that are partially or largely ‘uncoupled’ from the production of NO [109,132–136], but result instead in an excess of superoxide, H_2_O_2_ OONO^−^ and other RNIS [137]. Consequently, levels of NO may not be high enough for correct signalling, cytocidal and resolution purposes. iNOS may then get stuck, like a needle in a groove, chronically producing increasingly even less NO, and increasingly more dysregulating O_2_^−^, other ROS and RNIS. Such RNIS, produced in relatively modest amounts under normal conditions, play a very specific role in cell signal transduction and regulation of enzyme synthesis and degradation, by reversible covalent modification of proteins and enzyme systems [84–86]. Yet, if produced in increasingly excessive amounts, RNIS such as OONO^−^ can also affect the other principal cell signalling system, responsible for cell cycle control, tyrosine phosphorylation [138,139]. Nitration of tyrosine residues located near phosphorylation sites is irreversible, and impairs both the rate of phosphorylation and its reversibility [140], contributing further to unresolved inflammation.

This hypothesis also proposes that, in the absence of deficiency, Cbl may be the ultimate supplier of the substrates and cofactors necessary for efficient, more coupled, iNOS/nNOS NO production, as opposed to excess O_2_^−^, OONO^−^ and RNIS, and that it may consequently be the ultimate determinant of the NO/O_2_^−^ balance thought to be critical in achieving NO regulation of ONOO^−^-mediated signal specificity and response [86,136]. Moreover, Cbl, as GSCbl, may itself act as an additional direct promoter of iNOS, while simultaneously also acting as a ‘back-up disc’ to preserve or reactivate key antioxidant systems and enzymes that protect the host from damage during the immune inflammatory response. The resolution phase is ushered in by Cbl positively shifting the antioxidant balance, and having ensured effective, high levels of NO for a contained time span, so that NO eventually inhibits iNOS. A tantalizing clue to this proposed Cbl/NO relationship is to be found in the extraordinary capacity of liver for regeneration. In ancient mythology, Prometheus, the Titan who stole fire from Zeus for mankind, was punished by having a vulture devour his liver daily. Nightly, however, Prometheus's liver regenerated. This is scarcely myth: after resection of up to two-thirds of human liver, complete regeneration can occur within 2 weeks. During this period, iNOS is continuously active, as in foetal gestation. Might this powerful, safe and miraculous deployment of high NO over a long period of time have anything to do with the fact that the liver, with up to 5 years' supply, is the largest depository of Cbl in the body? Let us now consider the possible mechanisms.

## NOS: ‘in sickness and in health, for better or for worse’?

NO is produced by a family of NOS [136,141,142], heme-based enzymes that have a catalytic resemblance to cytochrome P450 and other heme-based oxygenases. NOS have been divided into three main classes: two constitutive forms, involved in respiration, cell signal transduction and neuro-transmission, respectively: membrane bound, particulate eNOS (NOS III), with a lipid anchor, targeted to the caveolae, the least active of the three isoforms, found primarily in smooth muscle and vascular endothelium; nNOS (NOS I), in the CNS/neurons and neuromuscular junctions; and iNOS (NOS II), produced in inflammatory immune responses by polymorphonuclear/granulocytes (PMN), primarily macrophages [81], the latter two enzymes both active in the cytosol. This rather simple picture has been complicated of late by the discovery of multiple isoforms and splice variants of NOS, and their discrete localization within subcellular compartments [86,136] – somewhat reminiscent of the varying forms of Cbl/TC distribution, localization and action. An example given is that of cardiac and skeletal muscle that coexpresses three different NOS isoforms in four different locations: a plasmalemal NOS, regulating force production and blood flow, a mitochondrial NOS, controlling respiration at the level of cytochrome *c* oxidase, a sarcoplasmic reticular NOS, involved in calcium homeostasis and other constitutive/inducible cytosolic NOS whose exact function/location had yet to be defined [143]. μ, α, β, γ tissue-specific isoforms of nNOS are also known [144]. Moreover, iNOS, hitherto thought to be active only briefly during immune responses, or chronically and aberrantly in pathologies of unresolved inflammation, has been shown to be continuously active, or constitutive, in B-lymphocytes [145], and in certain locations, such as the myocardium [146], retina [147], liver [148], bronchial epithelium [149], and also in murine ileum [150]. Since continuously expressed iNOS in the latter locations clearly has a positive protective function, removed from persistent pathology, (the lungs are continuously exposed to pathogens and iNOS NO in bronchial epithelium [151], yet most people do not have asthma, for example,) iNOS must be very tightly regulated, with strong endogenous safeguards to prevent its malfunction.

**Figure 3.**
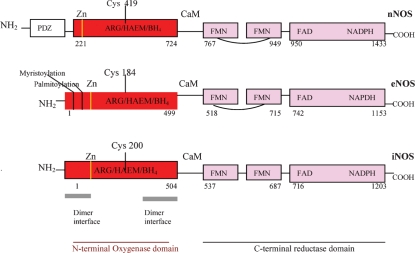
Human neuronal nitric oxide synthase (nNOS), endothelial NOS (eNOS) and inducible NOS (iNOS) domain structure. The PDZ domain is named after homologous domains in three proteins: PSD-95, DH/g, ZO-1.

The three human NOS have a 51–57% homology, and share a fundamental bi-domain structure: an N-terminal oxygenase domain, with binding sites for zinc, iron protophorphyrin IX (heme), L-arginine, (6R)-5,6,7,8-tetrahydrobiopterin (BH_4_), and a C-terminal reductase domain, with binding sites for the two flavins, FAD, FMN, and for NADPH. These two domains are linked by a calmodulin (CaM) recognition site (Figure 3). The crystal structures of the various NOS isoforms are gradually emerging [152–157], and the truncated iNOS oxygenase domain, elongated in form, with an unusual αβ fold, has been memorably described as like a ‘baseball catcher's mitt’, with the heme in the palm of the mitt [152]. Structurally, however, the NOS oxygenase domain differs from other heme oxygenases (cP450, peroxidase, catalase,) which have an α-helical distal pocket, whereas the NOS distal pocket has a β-sheet structure [136].

All three NOS monomers form dimers in their catalytically active states. Once the dimers are assembled, electrons donated by NADPH in the reductase domain are carried one by one by FAD to FMN and on to the oxygenase domain, where they reduce the heme iron and with BH_4_ supposedly catalyse oxidaton of L-arginine's terminal, N-guanidino via the intermediate, N^g^-hydroxyl-l-arginine (NHA) to NO and citrulline [136,141,142]. This is a two-step five-electron oxidation reaction. Ca^2+^/CaM is required for electron flow from the reductase to the oxygenase domains. However, whereas iNOS, which contains tightly bound CaM, under normal basal physiological conditions, can produce NO at low ambient Ca^2+^ concentrations, and is thus largely Ca^2+^ independent, nNOS and eNOS are totally dependent on cellular Ca^2+^ influx [136,141,142]. The amounts of NO produced by the constitutive and inducible isoforms also vary dramatically. Ca^2+^-dependent eNOS and nNOS produce NO continuously in short puffs and low amounts, with some modest up-regulation in inflammatory immune response, and up-regulation or depression in pathologies of unresolved inflammation, such as tumour growth or sepsis: Ca^2+^ independent iNOS, which is dependent for its expression on the transcription factors, NFκB, STAT-1, AP-1 and IRF-1 [141,158], can yield a 700–1000-fold greater increase in NO in a very short space of time.

We now come to another great point of controversy, which may be material to this hypothesis: whether, in fact, NO is a direct product of the NOS [109], or whether it is formed indirectly, after the formation of nitroxyl ion, NO^−^, or other RNI species, or s-nitrosothiols [141]. As with supposed iNOS over-activation and NO overproduction in sepsis, NO production by NOS in general has been deduced from the generic breakdown products, nitrite, nitrate, or effects on heme proteins such as soluble guanylate cyclase induction, or oxidation of oxyhaemoglobin to methaemoglobin [141]. Studies using specific NO electrodes or NO chemiluminescence assays have demonstrated that neither nNOS nor iNOS, at least, can produce NO in the absence of SOD, which reduces NO^−^ to NO [109,159,160]. SOD also, of course, scavenges O_2_^−^ produced by both FAD/FMN in the reductase domain [161,162], and in the oxygenase domains of eNOS/nNOS in the absence of BH_4_ [163,164]. Some evidence for NO^−^ formation in BH_4_ depleted iNOS also exists [165]. The general conclusion is that NOS may produce both NO and NO^−^ *in vivo*, in varying ratios, under different conditions [141]. The same is true of the NOS and O_2_^−^ production, which may be contained or excessive. Depletion of L-arginine, for example, increases NOS production of O_2_^−^, and subsequent H_2_O_2_ [166–168]. In the latter case, combinations of NO+O_2_^−^, or NO^−^ and O_2_, result in excessive ONOO^−^ and other RNIS [136,169], redundant to the normal physiological needs for ONOO^−^ and other RNIS for signalling/cytocidal purposes and thus likely to result in pathology. Such varying NO/NO^−^/ O_2_^−^ ratios will be determined by the redox environment, and, perhaps more critically, the availability of substrates and cofactors.

## BH_4_: the Scarlet Pimpernel's butterfly

The requirement for NOS enzyme activity of the cofactor BH_4_ (6R-5,6,7,8-tetrahydro-biopterin) is absolute [170]. The absence of BH_4_ results in no NO production, even with the L-arginine substrate [135]. Instead, BH_4_-free NOS catalyses a partially uncoupled NADPH L-arginine oxidation reaction, resulting in excess O_2_^−^ [164], and subsequent excess H_2_O_2_, and ONOO^−^. BH_4_-free murine NOS, expressed in *E. coli*, yields citrulline and N^δ^-cyanoornithine, nitrite and nitrate, but no NO [135]. (This is a good example of how these much-relied on ‘NO’ markers, nitrite and nitrate, may mislead.) Moreover, BH_4_ depletion is associated with vascular pathology [171]. Endothelial dysfunction due to eNOS inhibition, for example, has been reversed by BH_4_ administration to hypercholesterolaemic patients [172]. The addition of BH_4_ to eNOS increases NO production and decreases O_2_^−^ [173]. Furthermore, degrees of BH_4_ availability have been shown to limit the onset of iNOS NO synthesis, in a dose-dependent manner [135]. Conversely, the addition of exogenous BH_4_ to cells leads to earlier LPS-induced iNOS activity [170]. The absence of BH_4_ also appears to inhibit cell growth and differentiation via the inhibitionof NOS activity [174].

**Figure 4.**
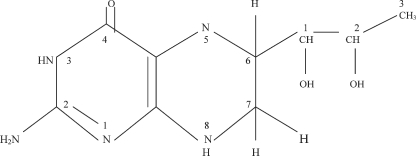
Structure of 6R-5,6,7,8-tetrahydro-L-biopterin (BH_4_), the naturally occurring pterin cofactor of nitric oxide synthases and amino acid hydroxylases. The standard nomenclature for numbering the positions of the pteridine ring and the biopterin side chain is indicated.

BH_4_ (Figure 4) is a pteridine, member of a class of pyrazino [2,3,d] pyrimidine compounds, which include molybdopterin and folate. Pteridines were initially discovered in the yellow pigments of the wings of butterflies by Sir Frederick Gowland Hopkins in 1889 [175]. The structures of three of these pigments were elucidated in the 1940s, thus giving rise to the name pteridine, from the Greek ‘ptera’ or wing. The isolation in 1957 of the structure of fluorescent blue pigments in the eye of *Drosophila melanogaster* [176], coincided with the discovery that the protozoan, *Crithidia fasiculata* required high doses of folic acid for growth and that biopterin could substitute wholly for it. Thus, it began to be appreciated that pteridines were not just pretty pigments, but had more complex biological functions, from light-gathering molecules to enzyme cofactors in redox reactions and I-carbon transfers [175]. Xanthine oxidase, for example, uses molybdopterin to catalyse the last step in purine synthesis [177]. BH_4_ is an essential reducing cofactor of aromatic amino acid hydroxylases (AAHs) in the metabolism of phenylalanine, tyrosine, tryptophan; epinephrine; the monoaminergic neurotransmitters: dopamine, norepinephrine, serotonin, and, en route to the latter, the sleep hormone, melatonin [177]. The observation of high BH_4_ levels in tissues low in AAHs, blood, spleen, lungs, has led to other proposed roles: a function of BH_4_ in haematopoietic cell proliferation and differentiation, based on the specific appearance of BH_4_ during the differentiation of reticulocytes to erythrocytes, and its cell cycle-dependent expression in various species, from mammalian thymocytes [178] to the acellular slime mould *Physarum*, in which BH_4_ levels peak during S-phase, and *Drosophila* embryos, which do not survive with BH_4_ mutation or inhibition [179]. The exact role of BH_4_ in the NOS has proven to be somewhat elusive and mysterious. It had been thought that it may have at least two functions: first, allosteric, stabilizing the NOS dimeric structure and the high-spin heme iron conformation; as well as promoting L-arginine substrate binding (which now appears not to be affected by BH_4_ [156]), second, a redox role as a direct electron donor to the heme, promoting conversion of L-arginine to the intermediate, NHA [153,156].

BH_4_, which is constitutive in liver, neuronal tissue and macrophages, can be additionally induced by LPS, or cytokines, principally, interferon-γ, TNFα and interleukin-1, in macrophages, fibroblasts, endothelial and vascular smooth muscle cells [175]. De novo BH_4_ synthesis requires the purine nucleotide, GTP, which is converted to BH_4_ in four enzymatic steps, by three separate enzymes [175]. GTP cyclohydrolase (GTPCH), which is rate limiting, catalyses the first step, yielding neopterin as a byproduct [175], a known marker of immune activation [180]. GTP availability increases the GTPCH reaction velocity in a concentration-dependent manner, and cytokine activation of cells significantly increases levels of GTP. The mechanism of this inflammatory-induced up-regulation is unknown [175].

## ‘Cherchez le Scarlet Pimpernel’: interferons and TCS to the rescue

Now, remember what happens to Cbl in inflammation? When NFκB is activated, it cross-talks to the transcription factor Sp1 [181], constitutively expressed in the eNOS promoter and known to be essential for NO production [141], and also expressed in the iNOS promoter region, along with the transcription factor IRF-1, essential for iNOS/eNOS mRNA expression and NO production [151]. Sp1 up-regulates the production of TCII [27], primed perhaps by interferons, particularly interferon-β, which simultaneously increases TCIIr expression [65] through further cross-talk between IRF-1 and Sp1. So more Cbl arrives intracellularly, where synthesis of GSCbl and its conversion to MeCbl and AdoCbl is up-regulated by decreasing cellular pH [38,42]. MeCbl binds to MS, which is then well armed to keep the folate pathway open and meet the increased needs in inflammation for purine nucleotide synthesis, essential for RNA synthesis and DNA replication. AdoCbl binds to MCoAM, and, as the cell's energy needs increase, AdoCbl, via MCoAM's impact on the TCA cycle and oxidative phosphorylation, ensures a steady stream of ATP, and the reducing equivalents, NADH+H^+^ and FADH_2_, for maximal efficiency of key defence enzymes. Each turn of the TCA cycle produces 1 mol GTP, and coupled to oxidative phosphorylation, 10 mol ATP [51]. ATP may be converted to GTP, and vice versa, but the ATP/GTP ratio in cells is relatively constant, with total cell concentrations of adenine nucleotides, ATP+ADP+AMP, being four to six times greater than those of guanine nucleotides, GTP+GDP+GMP. ATP and GTP are derived from AMP and GMP via the common ribonucleotide precursor in the de novo purine nucleotide synthesis pathway, inosine 5^1^-monophosphate (IMP), an energy expensive pathway in terms of ATP consumed per mole of IMP produced. The conversion of IMP to AMP and GMP is tightly and mutually regulated: with IMP to GMP requiring ATP, and IMPto AMP requiring GTP, as energy sources. So when there is sufficient ATP in the cell, GMP is synthesized from IMP, and when sufficient GTP exists, AMP is synthesized from IMP [177]. Thus, degrees of Cbl deficiency may ultimately impact on both levels of ATP and GTP, and increasingly depressed production of GTP will ultimately impact on BH_4_ status – IMP dehydrogenase inhibitors reduce BH_4_ synthesis [175]. Finally, in turn, this will inhibit iNOS NO production. A deficiency of BH_4_ will also ultimately affect its catalytic recycling by AAHs, resulting in decreased catecholamine and monoaminergic neurotransmitter synthesis [113]. Perhaps then the characteristic unresponsive hypotension of sepsis and septic or traumatic shock may be just as attributable to an increasing decline in catecholamine synthesis, as to the supposed overproduction of NO. Such LPS-induced hypotension has been shown to precede iNOS activation in rodents [182]. Furthermore, there may be an increase in oxidized and therefore ineffectual catecholamines such as adrenochrome [183] and adrenolutin, which also disturb mental equilibrium [184]. As Cbl is also critical to the synthesis of acetylcholine and the importance of the CNS and neuroimmune cholinergic pathway in inflammation has been well demonstrated [95,185], the characteristic mental obfuscation and loss of consciousness in sepsis [186] may also be as much consequences of depressed GTP synthesis as of supposed NO overproduction. Such a Cbl/GTP deficiency would result in a decrease in acetylcholine and other neurotransmitters, an increase in toxic catecholamine metabolites, and unregulated cytokine production. Indeed, given the low–high and vice versa NO/TNFα regulatory correlation discussed earlier, together with established high levels of TNFα in sepsis [187], and observed 83% increase in spinal fluid TNFα levels in Cbl deficiency [188], the argument for NO overproduction in sepsis looks tenuous, whereas it has been shown that higher levels of ATP in septic patients have a positive significant correlation with survival [189]. Declining ATP in sepsis would therefore correlate with a concomitant fall in levels of GTP, and consequently BH_4_ and consequently a decline in production of NO from iNOS, which would argue further against the popular view of increasing NO production in sepsis and other pathologies of unresolved inflammation.

## Cbl, αKG and arginine: close collaborators and distant allies

The production of succinyl CoA in the TCA cycle is not, of course, solely dependent on Cbl. α-Ketoglutarate, αKG, an important intermediate in the cycle, derived from citrate, is equally critical to succinyl CoA formation and the onward role of the cycle [51]. αKG is a form of ornithine, and its production is important not just for the activity of the TCA cycle, but for that of the urea cycle, the major mechanism for the removal of ammonia (NH_4_) produced by protein catabolism [113]. When αKG leaves the TCA cycle it is transaminated with glutamine to form glutamate, which can exit mitochondria and be further converted to other non-essential amino acids. In the CNS, αKG is converted to the neurotransmitters, glutamate and GABA, γ-aminobutyric acid. Glutamate can also be produced from αKG in mitochondria by the mitochondrial enzyme glutamate dehydrogenase in the presence of NADH or NADPH and ammonia [113]. The amino group thus incorporated into glutamine is used by aminotranferases to form other amino acids. Glutamate is a precursor of ornithine, in turn a precursor of arginine. In the urea cycle, which depends on a supply of glutamate, via αKG from the TCA cycle, ornithine and arginine are continuously interconverted, and the arginine formed there is not available for protein synthesis and NOS NO synthesis [113]. The synthesis of arginine for the latter begins in the intestinal mucosa, which is rich in glutamine and enzymes that convert glutamate via ornithine to citrulline. The final conversion of citrulline to arginine occurs in the renal proximal tubular cells, which lack the arginase of the urea cycle, and are responsible for 60% of net arginine synthesis [190]. Since citrulline is also a by-product of NO formation, most cells have the capacity to synthesize arginine to some degree, so that there is also in effect a citrulline– NO–arginine cycle, analogous to the recycling of arginine in the urea cycle [190]. Yet this recycling provides only about 50% of the L-arginine cofactor required by NOS to produce NO. The other 50% is supplied by arginine production from protein catabolism and synthesis [190]. Although no studies evaluate the role of the latter in regulating arginine availability for iNOS NO synthesis in different cell types, it is probably an important factor, as it is in arginine homeostasis, a balance kept by dietary arginine, endogenous arginine production and degradation [190]. In sepsis, where there is supposed iNOS NO overproduction, plasma arginine falls by as much as 50–60%, and the reason for this is not understood. Although high levels of iNOS/NO can inhibit protein synthesis, and inflammation induces both arginases and plasma arginine transporter up-regulation [190], thus potentially increasing plasma arginine clearance, there may be a different explanation, consistent with the hypothesis that in pathologies of unresolved inflammation, not enough NO is produced from NOS, but instead excess RNIS and ROS, so that the redox balance is lost, leading to increasing bioenergetic failure. High metabolic rates and increased catabolism without corresponding anabolism, characteristic of sepsis, mean that the liver and kidneys have to deal with the clearance of increasing levels of ammonia, the final breakdown product of protein catabolism. Glutamine, which constitutes 50% of circulating amino acids, and serves as an ammonia transporter, is diverted, as a result of lactic acidosis, from the intestinal mucosa to the kidneys – breakdown of the intestinal mucosa integrity is an early symptom of sepsis [191]. The uptake of glutamine by the liver, needed for the urea cycle, is suppressed and more is directed to the kidneys to conserve bicarbonate required for the formation of urea [113]. If the redox balance is not regained, persistent lactic acidosis and protein catabolism mean that the already dysregulated liver and kidneys start to fail. High ammonia concentrations lead to increasing liver sequestration of αKG as glutamate from the TCA cycle. This in turn reduces further the already reduced synthesis of ATP, leading to coma and death.

Thus, it is possible to see why there are two sources of succinyl CoA in the TCA cycle: Cbl functions as the back-up disc for αKG. If Cbl status is replete, fluctuations in αKG will be minimized, homeostasis of the TCA cycle ensured, in turn ensuring homeostasis of the urea cycle, and the availability of arginine outside the urea cycle for protein synthesis and NOS NO production. This is why the 5 g supra-physiological doses of Cbl used for 40 years by the French as an antidote to cyanide poisoning work so dramatically, restoring metabolism in hours and reversing damage to vital organs, so that liver function is normal at discharge, just 2 days after fatal doses of cyanide [66,72–76]. Cbl does this not just by binding to CN, but by performing all its normal metabolic tasks, which include the supply of all the NOS substrates and cofactors: the purine nucleotides, FAD and FMN, via MS; BH_4_ via GTP; arginine by supporting αKG and keeping the onward momentum of the TCA cycle rolling (consequently decreasing glycolysis and reversing lactic acidosis); NADPH, as we will see, via Cbl's impact on G6PDH and the pentose phosphate pathway; and last but not least, even heme synthesis, the organic portion of which requires eight residues each of glycine and succinyl CoA [192]. Cbl's critical role in heme synthesis particularly demonstrates the pivotal role Cbl may play in inflammation control, not only because many heme proteins such as cP450, catalase, HO-1, have anti-inflammatory functions, but because inducible HO-1 promoted by Cbl (USP 5,674,505), continually breaks down heme, via biliverdin, to the potent antioxidant, bilirubin. However, the simultaneous up-regulation of the TCs in inflammation ensures both the rapid regeneration of heme, and thus of key heme proteins, and a consequent increased potential production of bilirubin, as needed. (That AdoCbl via MCoAM is rate regulating in particular for the synthesis of the heme of haemoglobin has been specifically observed with heme staining of transparent 2 day old zebrafish larvae using o-dianisidine. T. Penberthy, pers. commun.)

eNOS is depressed in sepsis [107,193] and other pathologies of unresolved inflammation, and the evidence for alleged iNOS overexpression under these conditions is largely indirect or equivocal. iNOS may in fact be depressed as well as malfunctioning. Moreover, murine iNOS knockout models show that suppression of iNOS offers no protection against organ damage and mortality from LPS. iNOS expression and NO production are also dependent on the expression of interferons and the transcription factor, IRF-1, in the iNOS promoter. Might this be because interferons up-regulate TCS and their receptors, increasing and sustaining the arrival of more Cbl intracellularly, so that subsequently these increased local levels of Cbl, as AdoCbl and MeCbl, can ensure the assembly of all substrates and cofactors to enhance NO production, not only via iNOS, but eNOS, as seen in recent studies? [17].

## NO: friend or foe?

If it were not for the fact that it is a colourless compound, NO itself might well deserve the soubriquet ‘Scarlet Pimpernel’. Unknown until the last 25 or so years, the smallest but potent working mammalian molecule, NO, a paramagnetic gas, is produced enzymatically in diverse locations throughout the body and being both lipophilic and hydrophilic is easily diffusible towards its intracellular targets where exact specificity is achieved by the redox environment [64]. For example, in the ryanodine receptor/Ca^2+^ release channel (RyR), NO nitrosylates only one out of 50 free cysteines per RyR, to alter the Ca^2+^ CaM/RyR interaction, sensitizing the channel to positive or negative Ca^2+^ regulation [194]. This extraordinary precision can only happen at a restricted O_2_ concentration. NO, a second messenger for post-translational modification, is responsible for a wide diversity of critical functions: immune regulation and anti-microbial defence; neurotransmission and cerebral blood flow; smooth muscle relaxation; platelet aggregation or inhibition; exchange of gases in tissues; bronchodilation; glomerular filtration; gut peristalsis; penile erection; cardiac contractility; modulation of ligand-gated receptors (N-methyl-D-aspartate receptor) the Ca^2+^-dependent potassium channel, the cardiac Ca^2+^ release channel, cyclic nucleotide cation gated channels, Janus kinases, tyrosine phosphatases; peptide hormone release, and more [86,195–197].

To effect all of these physiological processes, NO assumes three redox forms, the free radical NO^·^ itself, nitrosonium (NO^+^) and the nitroxyl anion (NO^−^). So the term NO is a collective description. These varying redox forms of NO have particular affinities for particular biological targets: NO^·^ reacts with oxygen, superoxide O_2_^−^ and redox metals; NO^−^ with SH groups and metals; NO+ undergoes addition and substitution reactions with nucleophiles in aromatic compounds and electron-rich bases [64]. Fluctuations between these redox forms and their target products are of central importance to physiological homeostasis. Principally NO's strong affinity for transition metals results in its combination with the target heme iron, to form very stable catalytic Fe^2+^–NO iron–nitrosyl complexes, and/or combine with critical thiol residues, to form s-nitrosothiols, or s-nitrosylate proteins [64,86,138]. Such combinations can simultaneously activate some enzymes and deactivate others, modifying protein function or initiating gene expression. Both Cox I and II [138,198] and soluble guanylate cyclase and thence the second messenger cGMP [199,200] are activated by NO binding to their Fe^3+^, whereas cytochrome *c* oxidase, complex IV of the oxidative phosphorylation, electron transport chain, is deactivated by NO/Fe^3+^ binding [201] as are cP450 enzymes [202], indoleamine 2, 3-dioxygenase (IDO) [203], important in bacteriostasis, and, indeed, NOS itself [19–21]. Enzymes with catalytic thiols deactivated by s-nitrosylation include glyceraldehyde phosphate dehydrogenase, inhibited by NO-promoted ADP-ribosylation [204,205], protein kinase C [206], γglutamylcysteinyl synthetase [207], alcohol and aldehyde dehydrogenase [208], aldolase [208], cathepsin B [208], O^6^-methylguanine-DNA-methyltransferase [208], neutrophil NADPH oxidase [86,138] and at least seven members of the caspase family, involved in apoptotic signal transduction and cytokine maturation [86,209] and one of the most important antioxidant enzymes, GPX [210]. Conversely, s-nitrosylation of tissue plasminogen activator activates its vasodilatory and anti-platelet effects [211].

When the redox balance is maintained and oxidative stress is not extreme or persistent, such NO-derived enzymatic activation and deactivation will occur reversibly, in a complementary manner, both simultaneously and/or in a relay system, preventing NO toxicity. But persistent or extreme oxidative stress may render NO toxic by shifting the balance between NO redox species and their target reactants and end products, from high output of s-nitrosothiols, resistant to O_2_ and O_2_^−^ interaction, the principal form in which NO is neutralized for safe transport to tissues [64] via haemoglobin, myoglobin, albumin [212] and GSH [213–215], to increased production of RNIS such as the reactive intermediate, ONOO^−^, via increased reactions with H_2_O_2_ and O_2_^−^, and other ROS, which can prolong oxidative stress indefinitely, leading to increasing enzymatic malfunction, which will, of course, include malfunction of all three NOS.

If we assume that the NOS malfunction in extreme or persistent oxidative stress produces less NO and more O_2_^−^, then because the relative fluxes of NO and O_2_^−^ modulate the oxidation of critical enzymatic and other protein thiols by ONOO^−^ and other RNIS, and because ONOO^−^ and O_2_^−^ , rather than NO, inactivate aconitase [216] in the TCA cycle, and also inactivate NADH dehydrogenase and succinate dehydrogenase, complexes I, II and III of the oxidative phosphorylation, electron transport chain [217], as well as deregulating poly-ADP-ribosyl transferase [204,205], the stage is thus set for terminal cell function dysregulation and energetic failure. Of course, normally this does not happen due to powerful antioxidant defence systems, which are well primed and in place before the conveniently delayed induction of NO by iNOS peaking at 6 hours. But sometimes these systems fail, and when they do, this hypothesis posits, it is because they have lost their ‘back-up disc’, as a result of insufficient, or non-functional, Cbl status. Moreover, it seems that there is a fine hierarchical balance relationship between key anti-oxidant enzymes, which can be equally detrimental if disturbed, even though some of the enzymes may remain functional. With certain anti-oxidant enzymes more is not necessarily better. Indeed, it may be worse: five-and 10-fold increases in Mn-SOD and Fe-SOD, respectively, sensitize *E. coli* to paraquat toxicity [218,219]; Cu/Zn-SOD transfectants of mouse epidermal cells JB6 possess increased sensitivity to DNA strand breakage and growth inhibition in the presence of O_2_^−^ and H_2_O_2_ from xanthine/xanthine oxidase [220], whereas a concomitant increase in catalase in a double transfectant can correct this [220]. Similarly, in protection from ischaemia-reperfusion injury, a combination of Cu/Zn-SOD and catalase worked better than either alone [221]. Again, it has been observed in several systems that an increase in Cu/Zn-SOD is accompanied by an increase in GPX [222], with a high ratio of activity of GPX over Cu/Zn-SOD related to notably increased growth potential and resistance to killing by paraquat in NIH-3T3 transfectants [223]. Catalase scavenges H_2_O_2_ produced by Cu/Zn-SOD catalysis, but GPX, the primary H_2_O_2_ scavenger, additionally can destroy hydroperoxides, which catalase cannot, so the activity status of GPX is crucial and superior in the hierarchy, and ultimately that is dependent on the status of GSH, which apparently depends on the activity of GSH reductase, as well as the availability of cysteine, derived from serine, which provides the carbon skeleton, with supply of the sulphur dependent in turn on the reduction of homocysteine by MeCbl.

## GSH, Cbl and NO: an eternal triangle?

GSH, C_10_H_17_N_3_O_6_S, is actually a tripeptide, γ-glutamylcysteinylglycine, and so it has a less obvious debt to AdoCbl, via AdoCbl support of αKG in the TCA cycle, which ensures a supply of glutamate. An additional debt to MeCbl is in the supply of glycine, formed from the breakdown of serine in a reaction requiring pyridoxal phosphate and THF [113], the latter dependent on Cbl status for its availability [224]. As ubiquitous as Cbl and NO, GSH is a major reductant and detoxifier, involved in drug conjugation, and is a cofactor modulating O_2_^−^ production in many key enzyme systems, including glycolytic enzymes [91,214,225–227]. This function of GSH is of particular importance in the NOS. GSH is essential for leukotriene synthesis [113], amino acid transport [113], maintenance of erythrocyte membrane integrity [113], and reduction of peroxides formed during oxygen transport [91]. GSH is the principal thiol involved in the correct formation and degradation of protein disulphide bridges and through thiol–disulphide exchanges ensures proteins are folded into their native conformation [228]. Lysozyme, ribonuclease, albumin and insulin are notable examples of important proteins requiring GSH for formation [228]. GSH also ensures the reversibility of mixed disulphides GSSG, involved in the reactivity of proteins [228]. The functionally correct ratio of S-H groups to S-S is ensured by very high levels of GSH in cells, of up to 10 mM. In erythrocytes, for example, this results in a 100:1 ratio of GSH to GSSG [91]. Thus, the maintenance of very high levels of GSH, important in every respect, is critical for good immune function and the resolution of inflammation. GSH and GPX rapid depletion in sepsis has already been noted, a depletion that affects both arms of the immune system, with decreased lymphocyte response to mitogens in the relative absence of GSH [229]. Given the shift in redox balance entailed by the inflammatory immune response, the recycling of GSH by GSH reductase is liable to be ultimately inadequate without de novo GSH synthesis, and, as seen in pathologies of unresolvable inflammation, even this can fail. The GSH-sparing/promotional role of Cbl in preventing this appears to have been completely overlooked [1].

Yet, over 50 years ago it was observed that in pernicious anaemia/Cbl deficiency, in both rats and humans, levels of GSH in blood are very considerably depressed, and that these low levels return to normal promptly, and without exogenous GSH or GSH precursor administration, simply by treating the Cbl deficiency. Moreover, an initial overshoot of GSH was observed on correction of the Cbl deficiency [230,231]. That this is peculiar to Cbl was proved by studies showing that the normal reduction of S-S groups remains unaffected in iron or folic acid deficiency [231]. Contemporaneously, it was also shown that a combination of GSH and Cbl was synergistically more powerful than either alone in the reactivation of a range of enzymes with active S-H groups in the *E. coli* mutant 113–3 [232]. It was noted, however, that certain other enzymes were conversely deactivated by GSH alone [232]. These studies were, of course, performed in the era before the discovery of NO's biochemical role. With hindsight, given the NO-analogous effect of Cbl and GSH on enzyme activation/deactivation, one can see that these GSH/Cbl effects, mediated perhaps by possible Cbl oxidation and reduction of thiols [232–236], are part of bacterial defence against NO generated by macrophages in the phagocytic burst. In fact, as the studies used a combination of 50 mM GSH to 50 μg Cbl, it is likely perhaps that GSCbl was formed in the cultures. A noteworthy observation of these studies was that the GSH/ Cbl combination was most effective at regenerating activity in aged enzymes [232], thus simulating the effects of possible GSCbl on enzymes during oxidative stress, characteristic of inflammation, and again, with hindsight, perhaps indicating the potential importance of the controlled but increased rate of GSCbl formation in inflammation. Pertinently, enzymatic activity of G6PDH with added substrate was assayed in a range of pH, at 37°C, with Cbl and GSH, and found to be least active at pH 8.5, and most active at pH 6.5 – this is interestingly coincident with the pH variable for GSCbl's rate constant in inflammation. Most crucially from this viewpoint, the combination of GSH/Cbl was able to restore the activity of both lactate dehydrogenase and G6PDH [232], key enzymes in the pentose phosphate pathway, the latter responsible, with glycolysis and the TCA cycle, for the synthesis of the reducing equivalent, NADPH [94]. The activity of G6PDH is essential for the maintenance of erythocyte GSH in its reduced state via the formation of NADPH [94] and, of course, NADPH is essential to the function of key enzyme systems from cP450 to GSH reductase, GPX and the NOS. An irreversible decline in NADPH production during inflammation will have serious consequences, and will certainly result in increasingly less coupled NOS reactions and less NO. Other enzymes activated by Cbl/GSH include: pyruvic transaminase, stearic acid oxidase, glycine and alamine deaminase, cysteine desulphurase, serine dehydrase, maltase, lactase, lysine and ornithine and glutamic decarboxylase. [232] This last is of obvious significance for supply of αKG, arginine, glutamate, glutamine, etc. Moreover, the range of enzymes that responded suggested that this Cbl+GSH enzymatic activation was a general effect, not restricted to a particular class.

These early studies all provide forgotten evidence for an intimate co-dependent relationship between GSH and Cbl. The nexus of this co-dependence is in what has been described as a SAM ‘switch’ for the two alternating fates of homocysteine [127] (Figure 2). Homocysteine in the MeCbl MS-dependent methionine cycle is used to regenerate methionine, which reacts with ATP – from the AdoCbl MCoAM supported TCA cycle – to yield SAM, the universal methyl donor. If there is an excess of methionine, some of it will be converted to ammonia and α-ketobutyrate, which is decarboxylated to yield propionyl CoA [113] then converted to succinyl CoA and thence re-enters the TCA energy generation cycle. Some excess methionine will also be utilized in gluconeogenesis [113]. This shuttling of excess methionine back to the TCA cycle to repay the ATP debt for SAM shows the complementary, co-operative functions of the two Cbl coenzymes, everywhere apparent given the universal roles of MeCbl/AdoCbl, respectively, promoted by SAM and ATP. Levels of SAM are hence ultimately determined by Cbl status in general, as much as by dietary intake of methionine. If Cbl status is good, ATP and SAM will be abundant. High levels of SAM activate the SAM ‘switch’ that links Cbl to the synthesis of GSH, as SAM inhibits the provision of methyl folate for MS reduction of homocysteine to methionine, and increases cystathionine β-synthase, which transulphurates homocysteine to cystathionine, a precursor of cysteine and thence GSH [127].

This ultimate dependence of GSH status on Cbl status will have a direct impact on NO regulation because cellular thiol status has a major impact on NO species formation. The induction of iNOS is thus linked to the induction of GSH synthesis [237]. In the presence of thiol, s-nitrosation is preferential to O-or N-nitrosation [238], and, in health and a healthy immune response, s-nitrosothiols predominate over other nitrosated forms. Moreover, GSH and other thiols limit NO/O_2_^−^ interactions that produce excessive RNIS and ROS resulting in lipid peroxidation, excess S-H oxidation and other detrimental consequences [64,84,85,240]. For example, ONOO^−^ formed by NO/O_2_^−^ interaction can react with GSH to give s-nitroglutathione (GSNO_2_), which decomposes spontaneously to NO [241]. So the normal Cbl-GSH-dependent preponderance of RS-NOs over RNIS favours the positive aspects of NO regulation over the pathologies of NO dysregulation. The brilliantly host advantageous deployment in the Cbl–GSH–NO relationship of the potentially dangerous dual aspects of NO can be well illustrated by what happens in bacteriostasis/phagocytosis. Many bacteria contain similar defence systems to eukaryotes [86], such as SOD, GSH [242]/GPX and Cbl, in addition to an NO reductase, and G6PDH. During bacteriostasis and phagocytosis, however, neutrophils secrete large quantities of TCI, along with myeloperoxidase in the secondary granules. These TCIs act as magnets that leach Cbl from bacteria [243] (analogously to lactoferrin and iron), which means that bacteria are effectively disarmed, bereft of the back-up system that sustains their G6PDH and other enzymatic activity, in particular, their GSH/GPX, which is rapidly consumed in the increased acidity of the phagocytic burst and exposure to high levels of NO/ONOO^−^/O_2_^−^/H_2_O_2_ from the host. This lethal assault also affects the host's defences, with a 25% utilization of GSH in the first 10 min of phagocytosis [82]. However, the host is well armed to regenerate and synthesize GSH de novo, with significant amounts of AdoCbl on TCIs, in situ, not to mention incoming extra supplies from the phagocytosed pathogen. It has also been noted that s-nitrosothiols, such as GSNO, or s-nitrosocysteine, have a significantly more potent virustatic, parasiticidal, and bactericidal activity than NO itself, with trans-s-nitrosation implicated [81]. The regeneration of GSH, ultimately dependent on Cbl, is critical in this respect. One way or another it seems, the effects of NO cannot be subtracted from the effects of GSH and Cbl and when they are in balance, when Cbl status is sufficient, NO, GSH and Cbl may continuously regulate the NOS.
